# Anti-polyphenol oxidase properties of total flavonoids from young loquat fruits: inhibitory activity and mechanism

**DOI:** 10.1080/21655979.2021.1886387

**Published:** 2021-02-15

**Authors:** Gui-Li Huang, Ling-Xiang Sun, Jia-Jia Ma, Si-Yao Sui, Yu-Ning Wang

**Affiliations:** a Agricultural Product Storage and Processing Laboratory, Suzhou Academy of Agricultural Sciences, Suzhou, China; b Jiangsu Key Laboratory for Biomass Energy and Material, Institute of Chemical Industry of Forest Products CAF, Nanjing, China

**Keywords:** Polyphenol oxidase, total flavonoids, young loquat fruits, inhibitory mechanism

## Abstract

This study investigated anti-polyphenol oxidase activity and mechanism of purified total flavonoids (PTF) from young loquat fruits. PTF remarkably inhibited the activity of polyphenol oxidase (PPO) with an *IC*_50_ value of 21.03 ± 2.37 μg/mL. Based on enzyme kinetics, PTF was found to be a potent, mixed-type, and reversible inhibitor of PPO. The fluorescence intensity of PPO was quenched by PTF through forming a PTF-PPO complex in a static procedure. Therefore, this study authenticated PTF as an efficient PPO inhibitor, which would contribute to their utilization in food industry.

## Graphical Abstract

**Figure:**
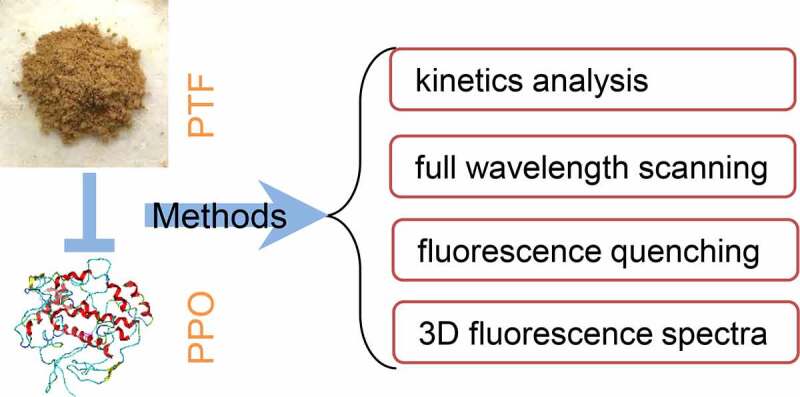

## 1. Introduction

Polyphenol oxidase (PPO, EC 1.14.18.1), or tyrosinase, is a rate-limiting enzyme in the catalysis of _L_-DOPA to o-quinones, and finally melanin biosynthesis [1,2]. In the food industry, PPO has a crucial role in the enzyme-mediated browning of vegetables and fruits, which often leads to significant losses of economic value and nutritional quality [3]. Browning due to enzymatic activities is a major problem of the food industry, particularly for vegetables, seafood products, and fruits [4]. Hence, efficient and secure inhibitors of PPO are crucial for their use in the food industry.

Browning in food results in a multitude of adverse effects on food preservation. Therefore, browning inhibitors are widely utilized in the food industry for the inhibition of browning due to enzymes [5,6]. Various anti-browning agents such as oxalic acid, citric acid, 4-hexylresorcinol, and ascorbic acid are good browning inhibitors, but they are not as effective as sulfite compounds which were confirmed potential health risks to sensitive individuals [7]. Therefore, it is imperative to discover effective and safe options for these chemicals.

Natural flavonoids have been receiving increasing attention due to their various biological activities. The flavonoids have been shown to be effective in the treatment of various morbidities such as cardiovascular risk, Alzheimer’s disease, age-related disorders, hyperglycemia, and cancer [8]. In particular, the flavonoids also have antibacterial, antioxidative, and anti-inflammatory effects [9]. A large number of young loquat fruits are clipped off during the process of planting to achieve better yield and quality of loquat fruits. So there are sufficient raw materials of the young loquat fruits for extraction of useful chemicals. However, there are only a few relevant studies on the flavonoids of young loquat fruits.

In this research, we investigated the inhibition effect and mechanism of PTF on PPO by using UV-visible absorption spectroscopy, full wavelength scanning study, fluorescence quenching assay, and three-dimensional fluorescence spectra. This study aimed to evaluate purified total flavonoids (PTF) from young loquat fruits and its anti-polyphenol oxidase activity and offer a scientific basis for their applications in food industry.

## 2. Materials and methods

### 2.1. Reagents and enzymes

L-3,4-dihydroxyphenylalanine (_L_-DOPA), polyphenol oxidase (EC 1.14.18.1), and Rutin were procured from Sigma-Aldrich (St. Louis, USA). Ethanol for the extraction was obtained from Sinopharm (Shanghai, China). Macroporous adsorption resin AB-8 was obtained from Donghong Chemical Co. Ltd (Jiangsu, China).

### 2.2. Extraction and purification of TF

The process of extraction and purification was implemented according to our previous report [10]. Young fruits of loquat of the ‘Baiyu’ cultivar were collected at Dongshan Zhen, Suzhou, in Jiangsu Province (China). The young loquat fruits were dried in an oven (DHG-9055A; Yiheng, China). Dry powder of the fruits was obtained by grinding and sieving the dried young fruits. The powders were extracted through ultrasound-assistance in a ratio of 1: 64 (solid: liquid; g/mL), in 49% ethanol at 65°C for 20 min. The crude flavonoids were then separated with an AB-8 column, which was eluted with water and ethanol-water (49%) in this order. Removal of ethanol was done through rotary evaporation and the part that remained was freeze-dried to acquire the purified total flavonoids (PTF). The standard used here was Rutin, and the estimated purity was 93.02%.

### 2.3. Wavelength scanning study

The study was conducted with reference to our previous experimental method [11]. _L_-DOPA oxidation was conducted in the presence or absence of PTF. Three milliliter of reaction media contained 0.5 mmol/L _L_-DOPA, sodium phosphate buffer at pH 6.8 and 20 μg/mL PTF. Finally, the enzyme was mixed into the media and the absorbance was measured on the Beckman DU-8000 spectrophotometer (Beckman Coulter, Pasadena, CA, USA).

### 2.4. Enzymatic kinetics experiments

The experiments were referred to previous studies method [12]. For the assay of enzyme activity, 3 mL of reaction media contained 0.5 mmol/L _L_-DOPA, sodium phosphate buffer at pH 6.8, and 0.1 mL PTF at different concentrations. The temperature of the reaction was 30°C. The enzyme activity was measured at 475 nm having 3700 (M^−1^ cm^−1^) as the molar absorption coefficient on the Beckman DU-8000 spectrophotometer.

### 2.5. Fluorescence quenching assay

The enzyme and inhibitor interaction was examined by fluorescence quenching [13], which was recorded by a Varian Cary Eclipse fluorescence spectrophotometer from Agilent (Santa Clara, USA) at 290 nm excitation wavelength [14]. The fluorophore of is Tyr and Trp residues. The fluorescence spectrum in the range of 300 to 450 nm was recorded. The kind of fluorescence quenching mechanism was determined using the Stern–Volmer equation [15].

$$F_{0}/F=1+K_{q}\tau_{0}\left[I\right]=1+K_{SV}\left[I\right]$$

where *F* and *F*_0_ indicate the intensities of fluorescence with or without an inhibitor. *K*_q_ denotes the rate constant for biomolecular quenching. τ_0_ represents the average fluorophore lifetime without the inhibitor (τ_0_ = 10^−8^ s). [I] denotes the inhibitor concentration. *K*_SV_ denotes the Stern–Volmer’s constant for quenching.

Dynamic and static are the two modes of fluorescence quenching [16]. For the static fluorescence quenching interaction, the calculation of *K*_a_ (binding constant) and *n* (number of binding sites) could be done by the double logarithmic equation [17].

$$log\left[\left(F_{0}-F\right)/F\right]\;=\;logK_{a}+nlog\left[I\right]$$

The PPO 3D (three-dimensional) fluorescence spectra with and without PTF were measured by using emission and excitation wavelengths in the range of 200 to 600 nm.

### 2.6. Statistical data analysis

Analyses of all statistical data were conducted using GraphPad Prism 6 (CA, USA). The error bars are represented as the means ± SEM from experiments performed in at least three triplicates.

## 3. Results

PPO is an important enzyme that is responsible for browning in fruits and vegetables. PPO inhibitors are quite important in the area of medicine and food industry. Natural products flavonoids have high therapeutic potential against multifactorial and complex diseases. In this study, the inhibitory effect and mechanism of PTF on PPO was studied by multi-spectroscopic methods. The results demonstrated that PTF was an effective inhibitor of PPO, which could provide scientific basis in the development of natural PPO inhibitors.

### 3.1. Impact of PTF concentration on PPO activity

**Figure 1.**
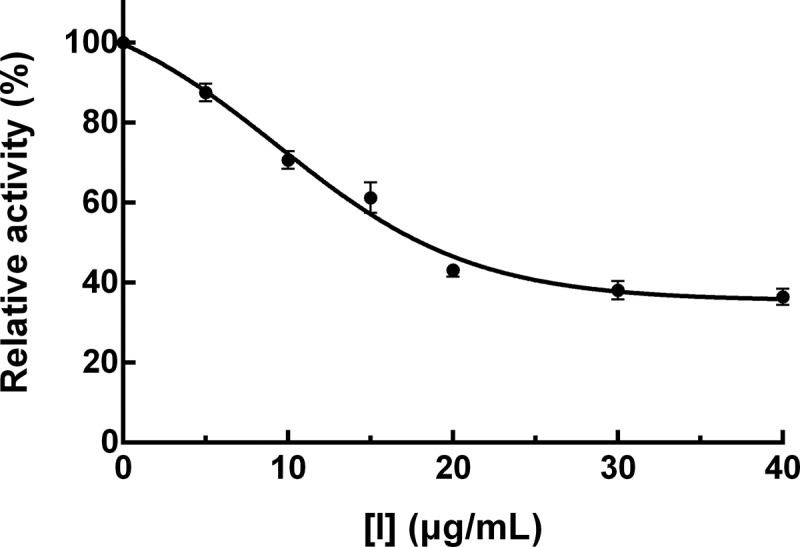
Inhibitory effect of PTF from young loquat fruits on PPO

The inhibition of PPO activity due to PTF from young loquat fruits was examined. As shown in Figure 1, PTF exhibited a strong inhibition against the PPO activity in a dose-dependent manner but was not suppressed completely. The concentration of PTF causing the loss of 50% relative enzyme activity (*IC*_50_) was determined to be 21.03 ± 2.37 μg/mL and was less than that of quercetin (*IC*_50_ = 39.2 ± 4.1 μg/mL) and proanthocyanidins from cherimoya pericarp (*IC*_50_ = 37.3 ± 3.6 μg/mL) [18,19], manifesting that PTF from young loquat fruits was a more effective PPO inhibitor.

### 3.2. _L_-DOPA oxidation in the absence and presence of PTF

**Figure 2.**
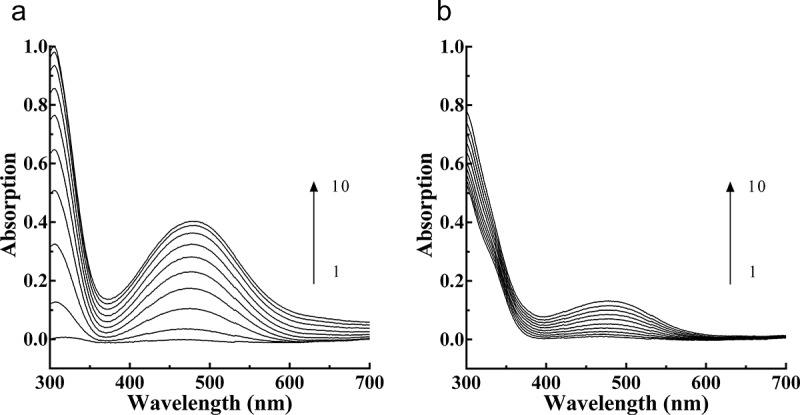
Consecutive spectra obtained during the oxidation of L-DOPA by PPO. (a), Consecutive spectra in the absence of PTF. (b). Consecutive spectra in the presence of PTF. Lines 1–10 represent 0–9 min after the addition of the enzyme. PTF: 20 μg/mL

The PPO catalysis spectra obtained during _L_-DOPA oxidation without (Figure 2(a)) and with (Figure 2(b)) PTF in the reaction system are presented. The peak absorbance at 475 nm was 0.402 at 9 min post-PPO addition. In the presence of PTF, the peak intensity decreased to 0.131, representing a decrease of 67.41%, indicating that PTF from young loquat fruits was a strong inhibitor of PPO.

### 3.3. PTF type and associated mechanism of PPO inhibition

**Figure 3.**
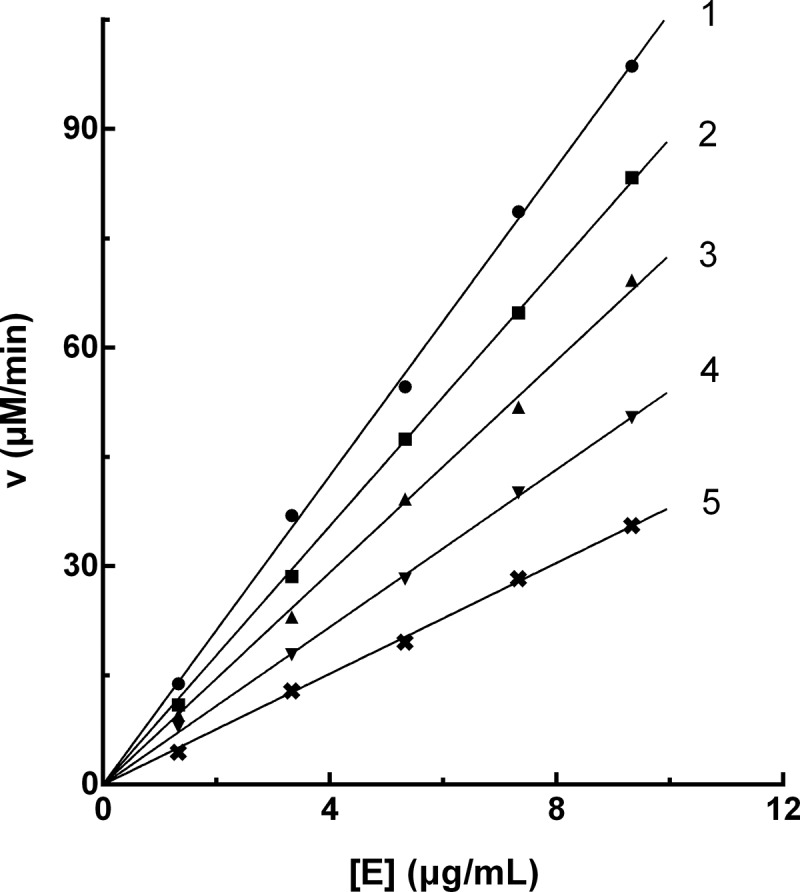
Inhibition mechanism of PTF from young loquat fruits on PPO. The concentrations of PTF for lines 1–5 were 0, 5, 10, 15, and 20 μg/mL

The enzyme activity against the PPO concentration with different concentrations of PTF in the reaction system was plotted to evaluate the inhibitory mechanism. As shown in Figure 3, a group of straight lines crossed the origin, and the increase in PTF concentration, caused a decrease in the slope of the line, showing that the presence of PTF did not reduce PPO content, but caused a decline in the activity of PPO. The above results suggested that the inhibition of PTF on PPO was reversible.

**Figure 4.**
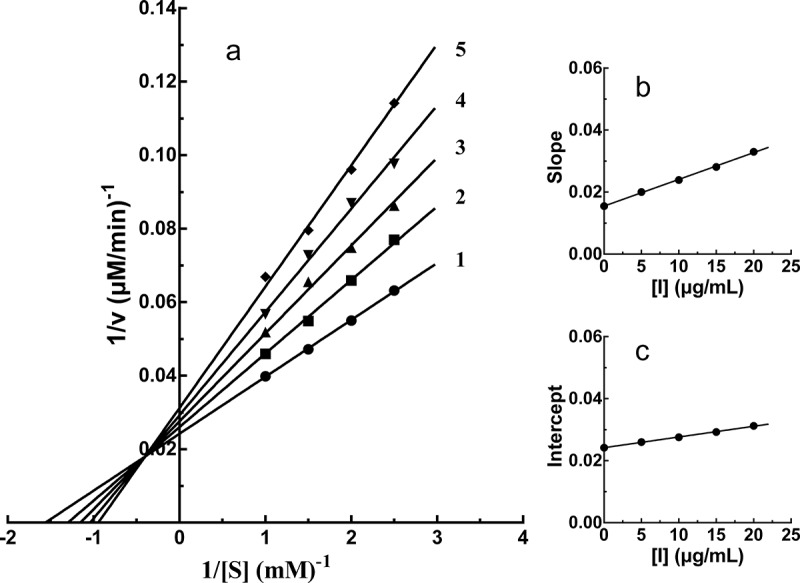
Inhibition type and inhibition constants of TF from young loquat fruits on PPO. The concentrations of TF for lines 1–5 were 0, 5, 10, 15, and 20 μg/mL

The inhibitory kinetic behavior of PTF on PPO was then studied. The Lineweaver-Burk plot was shown in Figure 4(a). The intersection of all straight lines occurred in the second quadrant, indicating the mixed-type inhibitor characteristic of PTF. The inhibition constants of PTF binding with the free enzyme (*K*_I_) and with the enzyme-substrate complex (*K*_IS_) were 17.95 ± 0.53 μg/mL and 69.99 ± 4.52 μg/mL, respectively. *K*_I_ and *K*_IS_ were calculated from Figure 4(b,c), respectively, with absolute value of intercept from X-axi. The value of *K*_IS_ was higher than that of *K*_I_, suggesting the affinity of PTF with PPO-_L_-DOPA complex was weaker than that with PPO.

### 3.4. PPO Fluorescence quenching in the presence of PTF

**Figure 5.**
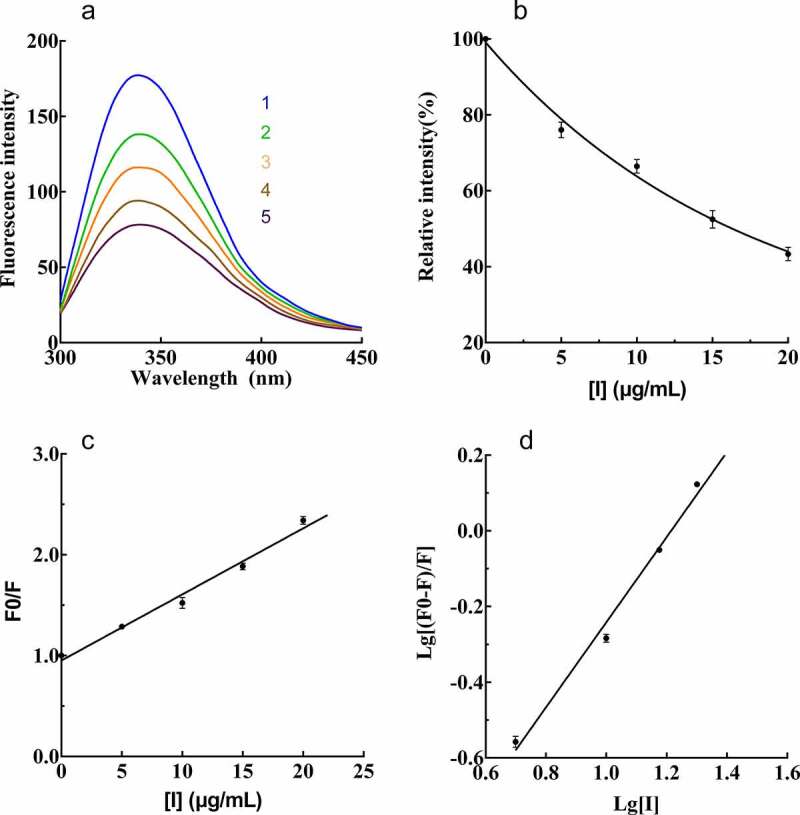
Effect of PTF from young loquat fruits on the emission spectrum of PPO. (a), Intrinsic fluorescence changes. (b), Florescence intensity changes. (c), Stern–Volmer curves of PTF. (d), Plot of log[(F0 − F)/F] against log[I] for PTF

To demonstrate the interaction between PPO and PTF, fluorescence quenching was carried out. As presented in Figure 5(a), there was a gradual reduction in fluorescence intensity of PPO with the increase in PTF concentration. The reduction was 56.66% when the PTF concentration was up to 20 μg/mL (Figure 5(b)), which demonstrated that PTF was a good fluorescence quencher of PPO.

The Stern-Volmer equation was applied to assess the mechanism of fluorescence quenching. F_0_/F increased gradually with increasing concentration of PTF (Figure 5(c)), which presented a good linear relationship, showing that the PPO fluorescence quenching process by PTF was in a static mode [20]. The values of *K*_q_ (biomolecular quenching rate constant) and *K*_SV_ was determined to be 5.578 × 10^6^ (μg/mL·s)^−1^ and 5.578 × 10^−2^ (μg/mL·s)^−1^, respectively.

For the static fluorescence quenching, *n* (the number of binding sites) and *K*_a_ (the binding constant) could be estimated from the lg[(F_0_ − F)/F] plot compared with lg[I] (Figure 5(d)). The values of *n* and *K*_a_ were 1.124 and 0.043 (μg/mL)^−1^, respectively. The number of binding sites (*n*) was nearly equal to 1, demonstrating only a single binding site for PTF on PPO.

### 3.5. Three-dimensional fluorescence spectroscopy

**Figure 6.**
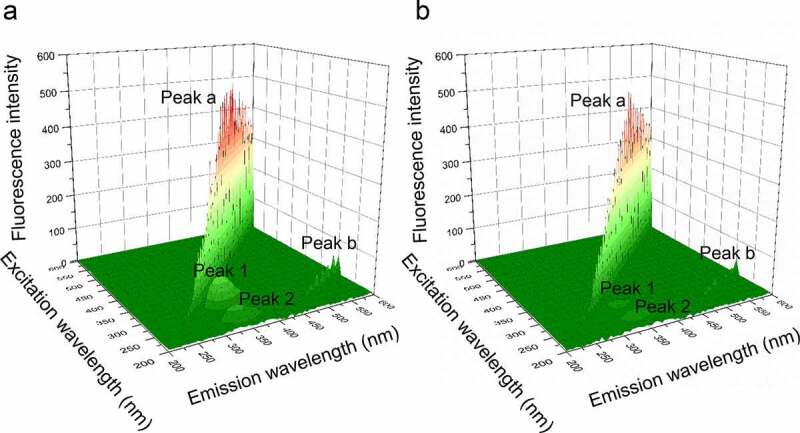
(a), Three-dimensional fluorescence spectra of PPO in the absence of PTF; (b), Three-dimensional fluorescence spectra of PPO in the presence of PTF (20 μg/mL)

A powerful fluorescence analysis assay, three-dimensional fluorescence spectroscopy, was used to investigate the conformational change of PPO. According to Figure 6, the peak a is the Rayleigh scattering peak (λ_ex_ = λ_em_), while peak b is the second-order-scattering peak (λ_ex_ = 2λ_em_) [21]. Peak 1 (λ_em_ = 340 nm, λ_ex_ = 280 nm) indicates the feature of Tyr and Trp residues. Peak 2 (λ_em_ = 340 nm, λ_ex_ = 230 nm) mainly reveals the characteristic of polypeptide chain skeletal structure of PPO, which caused by the π → π* transition [22]. With the addition of PTF to PPO solution, the intensity of fluorescence of peaks 1 and 2 reduced from 90.3 to 33.8, and 43.9 to 23.2, respectively. The above result demonstrates the conformational change of PPO during the formation of the PTF-PPO complex.

## 4. Discussion

The main catalytic function of PPO is conversion of tyrosine into melanin pigments, which leads to browning of vegetables and fruits [23]. Hence, efficient and secure inhibitor of PPO is crucial in improving food quality. Flavonoids are a broad group of low-molecular-weight natural antioxidant compounds [24]. In our present study, we found that natural extract PTF was an efficient inhibitor of PPO with the semi-inhibitory rate (*IC*_50_) 21.03 ± 2.37 μg/mL. From Lineweaver-Burk plot, we can calculate that the inhibition constants *K*_I_ was 17.95 ± 0.53 μg/mL and *K*_IS_ was 69.99 ± 4.52 μg/mL. The *K*_I_ value was much lower than the *K*_IS_ value, indicating that the inhibitor has a stronger affinity to PPO than PTF-PPO complex.

## 5. Conclusions

In conclusion, this study showed that PTF from young loquat fruits was a reversible, mixed competitive, and efficient inhibitor of PPO. The *IC*_50_ for PTF was 21.03 ± 2.37 μg/mL. The fluorescence of PPO was quenched by PTF due to PTF-PPO complex formation in a static process. In addition, PTF was bound to PPO at a single binding site and caused the conformational change of PPO. This study reveals the feasibility of using PTF from young loquat fruits in food industry.

## Supplementary Material

Supplemental Material

## Highlights

1. PTF is efficient, reversible, and mixed competitive inhibitors of PPO.
2. PTF has one binding site with PPO.
3. PTF induced conformational change of PPO.
