# Supplementary figures and images for: Anti-polyphenol oxidase properties of total flavonoids from young loquat fruits: inhibitory activity and mechanism
Source: Bioengineered. 2021 Feb 15;12(1):640–7. doi: 10.1080/21655979.2021.1886387 (PMC8806263; doi:10.1080/21655979.2021.1886387)

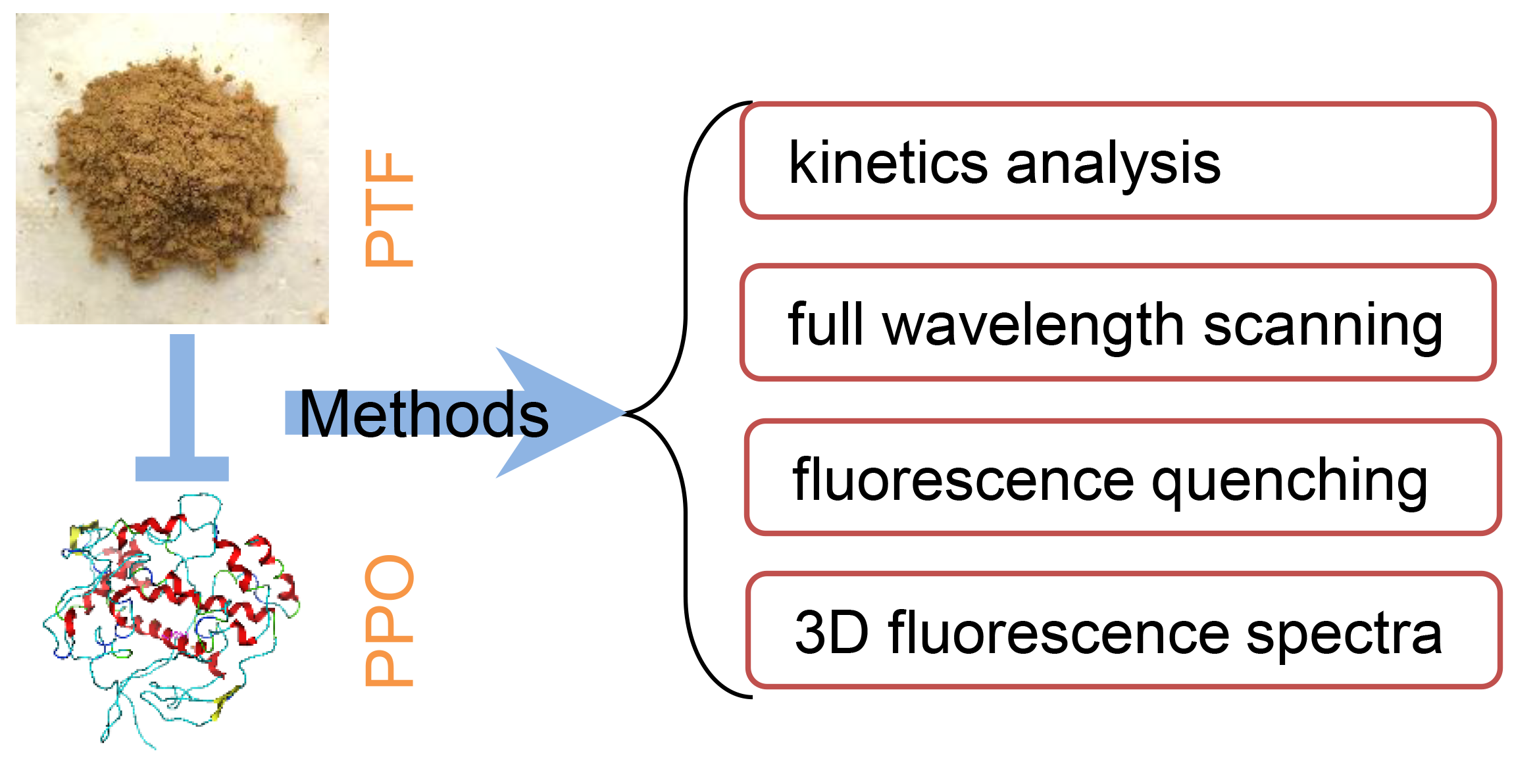

Supplement: Supplemental Material [file KBIE_A_1886387_SM7223.tif]
